# Comparative efficacy of sleep positional therapy, oral appliance therapy, and CPAP in obstructive sleep apnea: a meta-analysis of mean changes in key outcomes

**DOI:** 10.3389/fmed.2025.1517274

**Published:** 2025-02-03

**Authors:** Yunjun Gao, Sixiang Zhu, Wenjun Li, Youqing Lai

**Affiliations:** Department of Otorhinolaryngology, Beijing Jishuitan Hospital, Capital Medical University, Beijing, China

**Keywords:** obstructive sleep apnea, sleep positional therapy, continuous positive airway pressure, oral appliance therapy, systematic review

## Abstract

**Background:**

Obstructive sleep apnea (OSA) is commonly treated with continuous positive airway pressure (CPAP), though many patients struggle with adherence. Sleep positional therapy (SPT) offers a potential alternative, especially for positional OSA (POSA). This study aimed to compare the efficacy and safety of SPT with CPAP, oral appliance therapy (OAT), and placebo.

**Methods:**

Nineteen randomized controlled trials (RCTs) with 1,231 participants were included. Data extraction focused on changes in key outcomes such as apnea-hypopnea index (AHI), total sleep time (TST), oxygen desaturation index (ODI), and sleep architecture from pre- to post-intervention. Random-effects meta-analyses were conducted to compare mean changes between SPT and control groups (placebo, OAT, CPAP), with sensitivity analyses to assess heterogeneity.

**Results:**

Sleep positional therapy (SPT) showed a significant reduction in AHI in the supine position compared to placebo (MD = −7.46, 95% CI: −11.42, −3.49), although no difference was observed in overall AHI between SPT and placebo or OAT. Compared to CPAP, SPT was less effective in reducing AHI, with a trend toward greater reductions in AHI favoring CPAP. SPT demonstrated a significant improvement in arousal index compared to OAT (MD = −7.11, 95% CI: −10.52, −3.71) and a lower risk of device-related complications compared to both OAT (OR = 0.54, 95% CI: 0.31, 0.95) and CPAP (OR = 0.29, 95% CI: 0.12, 0.72). However, SPT did not lead to significant improvements in TST or oxygen saturation parameters across comparisons.

**Conclusion:**

Sleep positional therapy (SPT) is a safe alternative for managing positional OSA, particularly for patients intolerant to CPAP, though it remains less effective than CPAP in reducing overall AHI and improving oxygenation.

## 1 Introduction

Obstructive sleep apnea (OSA) is a prevalent sleep disorder characterized by repetitive episodes of partial or complete upper airway obstruction during sleep, leading to intermittent hypoxia, fragmented sleep, and a host of adverse health outcomes including cardiovascular disease, metabolic dysfunction, and impaired cognitive function (1). The standard of care for OSA management typically involves continuous positive airway pressure (CPAP) therapy, which has been shown to effectively reduce apnea-hypopnea index (AHI) and improve daytime symptoms (2, 3). However, adherence to CPAP remains suboptimal, with many patients discontinuing its use due to discomfort and inconvenience (4, 5).

Recent evidence has highlighted the intricate interplay between OSA and systemic inflammation, driven largely by oxidative stress as a result of intermittent hypoxia. This cyclic hypoxia triggers the overproduction of reactive oxygen species (ROS), leading to cellular damage and an imbalance in antioxidant defenses (6). These mechanisms exacerbate systemic inflammation, with elevated markers such as interleukins (IL-6 and IL-8) and tumor necrosis factor-alpha (TNF-α), contributing to cardiovascular, metabolic, and neurodegenerative comorbidities (6). These findings underscore the potential of targeting oxidative stress as a therapeutic strategy to mitigate the multifaceted impacts of OSA.

In recent years, alternative therapeutic approaches have been explored, including positional therapy, which aims to prevent the supine position during sleep—a known risk factor for exacerbating OSA severity (7). Positional obstructive sleep apnea (POSA) is defined as OSA that is significantly worse when the patient is in the supine position compared to lateral positions (8). Various positional therapy devices, ranging from simple techniques like tennis balls sewn into the back of sleepwear to more sophisticated devices such as vibrating alarms and specialized pillows, have been developed to address this issue (9).

Several meta-analyses have explored the efficacy of these alternative therapies (10–14), comparing CPAP, SPT, and OAT across various outcomes, including apnea-hypopnea index (AHI) reduction and sleep architecture. However, many of these reviews provide inconsistent conclusions regarding the relative efficacy of SPT compared to other therapies. A key limitation in prior analyses is their reliance on post-intervention comparisons without accounting for baseline differences between study groups. This approach may obscure the true impact of each therapy on patient outcomes.

In contrast, the present systematic review and meta-analysis aims to address these limitations by employing a more nuanced analytical method that focuses on the mean change in key outcomes from pre-intervention to post-intervention. This approach not only provides a clearer picture of how each therapy impacts OSA symptoms but also offers more clinically meaningful conclusions for practitioners seeking alternatives to CPAP. Additionally, we provide a comprehensive analysis across all parameters including sleep measures, respiratory indices, and other clinical outcomes.

## 2 Methods

### 2.1 Study design

This systematic review and meta-analysis was conducted following the Preferred Reporting Items for Systematic Reviews and Meta-Analyses (PRISMA) guidelines which was updated in 2020 (15).

### 2.2 Literature search

To identify eligible studies, PubMed, Scopus, Web of Science, Cochrane Central Register of Controlled Trials (CENTRAL), clinicaltrials.gov, and Google Scholar (only the first 200 citations were selected) (16), were searched from inception to 4 June 2024, as outlined in Supplementary Table 1. Citations were filtered based on their titles and abstracts. No restrictions were applied regarding the original language of publication. To ensure the accuracy of the performed search and screening, we searched for relevant studies manually by reading the reference list of finally selected papers, by checking the list of “similar articles” to selected ones on PubMed, and by manually searching Google software using the same keywords included in the literature search (17).

### 2.3 Selection strategy

The eligibility criteria were based on the refined PICOS (Population, Intervention, Comparison, Outcomes, and Study Design) framework (18). Selected studies followed this criterion:

1. Randomized controlled trials (study design).
2. Including patients diagnosed with OSA based on the Apnea-Hypopnea Index (AHI) or other sleep study parameters (population).
3. Patients receiving any form of positional therapy (intervention).
4. Comparing positional therapy to any other intervention, such as placebo (ad libitum; no treatment, general advice, supine position, standard pillows, or inflated airbags), oral appliance therapy (OAT), or CPAP (comparison/control group).
5. Reporting pre- and post-intervention data on relevant outcomes (listed below).

Alternatively, studies meeting the following criteria were ruled out:

1. Non-original research (i.e., reviews, editorials, perspectives, commentaries, etc.).
2. Non-randomized studies of intervention (experimental) and observational studies.
3. Studies including mixed populations (OSA plus other conditions) without stratifying their data based on studied disease or including patients with other conditions.
4. Trials investigating other interventions other than positional therapy.
5. No clear description of the intervention or comparison groups.
6. Duplicates studies.
7. Studies with overlapping patients’ data (confirmed by similar recruitment period, study settings, and patients’ baseline data).

### 2.4 Data collection and outcome measures

The senior author designed the data collection sheet using Microsoft Excel. The sheet was divided into three sections. The first one contained data pertaining to included studies (authors’ names, country, year of publication, study design, and follow-up), examined patients (population description, sample size, age, gender, exclusion criteria, and OSA severity at baseline), and administered interventions (types, name, and descriptions of allocated interventions).

The second part contained our outcomes of interest. The primary endpoint was AHI (total, supine, or non-supine position). Secondary outcomes included sleep parameters, respiratory indices, and other clinical outcomes. Sleep parameters included total sleep time (TST, min), which included the time of all sleep stages combined, including stage 1 (N1), stage 2 (N2), stage 3 (N3), and rapid eye movement (REM) combined. It also included % TST in SaO_2_ < 90% (known as CT90). Respiratory indices included sleep efficiency (%), arousal index, O_2_ desaturation index (ODI), and mean / minimum SaO_2_. Other clinical parameters included rate of treatment response (defined as reduction in AHI by at least 50% with supine TST score < 10), device-related complication rate, Epworth Sleepiness Scale (ESS), Functional Outcomes of Sleep Questionnaire (FOSQ), and persistent apnea or snoring or tiredness.

The third part covered the risk of bias assessment of included trials. Investigators were blinded to studies’ country and year of publication as well as authors’ names to minimize the risk of judgment bias. Each study was given an ID in this regard which was cross-validated with original study title by the corresponding author. Two authors were blinded to other investigators’ work and their role was to ensure the accuracy of the extracted data. In instances where inaccurate data or inconsistent reporting was found, a meeting with the corresponding author was done to correct these mistakes.

### 2.5 Risk of bias assessment

The risk of bias (RoB) of included studies was examined using the 2019 revised Cochrane RoB-2 tool. Each RCTs was assessed over several aspects: randomization, deviations from intended interventions, missing outcome data, selection of reported results. Each domain is given a rating of either “low risk,” “high risk,” or “some concerns” (19). Overall, if a study had high risk in one domain, it was designated as having an overall high risk of bias. If a study had low risk in all domains, it was designated as having an overall low risk of bias. Otherwise, the study was designated an overall rating of some concerns.

### 2.6 Statistical analysis

Before conducting any statistical tests, proper data handling was ensured. For studies reporting the median (IQR or range), data were transformed to mean (standard deviation—SD) using validated equations (20–23). Given the standardization of measurement scales, there was no need to calculate a standardized mean difference.

All statistical analyses were carried out using STATA Software (Version 18, Stata Corp, United States). In contrast to the standard meta-analytic approach that compares post-intervention results directly between intervention and control groups, this study calculated the mean change in each continuous outcome (e.g., AHI, ODI, TST) for each group (intervention vs. control) from pre-intervention to post-intervention. The mean change for each outcome was then compared between groups using random-effects meta-analyses. This approach allows for a more direct evaluation of the impact of the interventions on outcome improvements relative to baseline.

Random-effects meta-analyses were conducted to pool the mean differences (MD) in outcomes between the intervention (SPT) and control groups (placebo, OAT, and CPAP). The random effects model was employed to account for expected heterogeneity among included studies due to differences in study populations, intervention protocols, and outcome measures. This approach allows for the assumption that the true effect size may vary across studies, which is appropriate given the diversity of clinical settings and methodologies in the included studies.

The pooled mean difference (MD) and 95% confidence intervals (CI) were calculated for each comparison. Heterogeneity across studies was assessed using the I^2^ statistic, with values over 50% indicating substantial heterogeneity (24). Sensitivity analyses were conducted by removing individual studies to examine their impact on overall results. Subgroup analyses were performed to explore the effects of SPT device type, follow-up duration, and OSA severity on treatment outcomes, only if sufficient number of studies were available (at least five studies) (25). We inspected Galbraith plots to identify any outliers, and if any were found, the respective data were checked for accuracy and was then excluded if it ascertained to be accurate. The assessment of publication bias was not feasible due to the lack of enough power (< 10 studies per analysis).

Missing data were addressed as follows: studies with incomplete reporting of key outcome measures were excluded from the quantitative synthesis if sufficient data for imputation were not available. When possible, missing standard deviations were estimated using reported confidence intervals, standard errors, or other statistical measures as recommended by the Cochrane Handbook for Systematic Reviews (26). Sensitivity analyses were performed to assess the impact of missing data on overall effect sizes.

## 3 Results

### 3.1 Literature search results

**FIGURE 1:**
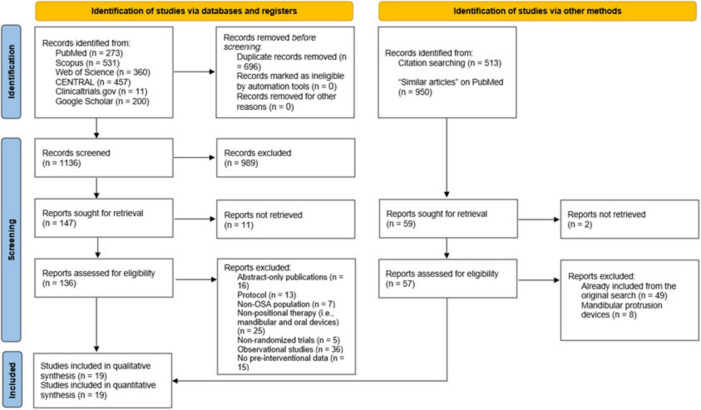
A Preferred Reporting Items for Systematic Reviews and Meta-Analyses (PRISMA) flow diagram showing the results of the database search and screening processes.

We retrieved 1,832 citations from the database search, of which 696 duplicates were ruled out by EndNote Software (Figure 1). The initial screening of 1,136 records yielded 147 articles eligible for full-text screening. Eleven studies were unretrievable, and from the remaining 136 studies, 117 were excluded for the following reasons: abstract-only publications (*n* = 16), study protocols (*n* = 13), non-OSA populations (*n* = 7), non-positional therapy like mandibular protrusion devices (n = 25), non-randomized studies of intervention (*n* = 5), observational studies (*n* = 36), and lack of pre-interventional data (*n* = 15). The manual search did not add any additional studies, resulting in 19 RCTs eligible for data synthesis and analysis (20–23, 27–41).

### 3.2 Baseline characteristics of included studies

A summary of included studies’ characteristics is provided in Table 1. Of included trials, nine were cross-over RCTs while the remaining studies were parallel RCTs. Eight RCTs compared SPT to placebo, five compared SPT to OAT, and six compared SPT to CPAP. Most RCTs were conducted in the United States (*n* = 4) followed by the Netherlands (*n* = 3), Australia (*n* = 2), and New Zealand (*n* = 2), respectively. A total of 1,231 OSA patients were examined, of whom 649 were in the SPT group, 205 in the placebo group, 222 in the OAT group, and 155 in the CPAP group.

**TABLE 1:** Baseline characteristics of randomized controlled trials of sleep positional therapies in patients with obstructive sleep apnea.

Author (YOP)	Population	Design	Country	Group	Description	Sample	Age (year)	Gender	FU (mo)	OSA severity		
								**M/F**		**Mild**	**Mod**	**Sev**
**SPT Versus Placebo**												
Armas (27)	POSA as having an AHI ≥ 5 per hour; supine AHI ≥ 2than in the non-supine position; TST ≥ 180 min; and TST in thesupine position ≥ 20% of TST.	Multicenter, parallel RCT	Spain	Placebo	General recommendations	41	51.3 (10.5)	34/7	3	–	–	–
				Active device	Somnibel positional therapy device and box of breathable fastening adhesive. The device was placed on the patient’s forehead.	43	53.4 (12.7)	34/9		–	–	–
				Inactive device	Somnibel (inactive)	44	51.8 (11.5)	35/9		–	–	–
Jackson (31)	Participants at least18 years of age, supine OSA (supine AHI at least twice the nonsupine AHI) on overnight diagnostic PSG, total AHI ≥ 10, and at least4 h of sleep with at least 30 min sleep in both the lateraland supine recumbent positions and 30 min of REM sleep	Parallel RCT	Australia	SPMD	A band of stretch cotton worn around the chest just below the nipple line. The band was situated at the front with buttons and the ball was contained.	47	48 (11.2)	37/10	1	–	100%	–
				Placebo	Sleep hygiene advice	39	51.2 (11.4)	30/9		–	–	–
Laub (33)	Consecutive patients diagnosed with POSAS were potential candidates for this study	Parallel RCT	Denmark	SPT	NightBalance, a small device placed in a pocket of a neoprene strap attached to the patients’ chest.	52	50.3 (12.9)	39/13	2	100%		–
				Placebo	No treatment	49	51.2 (13.3)	38/11		100%		–
Lukachan (34)	Adult patients, American Society of Anesthesiologists (ASA) physicalstatus I to IV, undergoing elective inpatient non-cardiacsurgery with newly diagnosed OSA.	Parallel RCT	USA	Semi-upright position	Head-end elevation 30-45 degrees from horizontal	21	65 (12)	7/14	7	–	–	–
				Placebo	Supine position	14	63 (10	9/5		–	–	–
Svatikova (23)	Patients with OSA and ischemic stroke	Cross-over RCT	USA	PT	SONA Pillow (a flat base and a double incline on the top surface that promotes lateral positioning	9	61 (13.5)	5/4	3	100%		–
				Placebo	Standard hospital pillow	9	57 (11.9)	6/3		100%		–
Zaremba (41)	Women with OSA who gave birth without adverse events	Cross-over RCT	Germany	Intervention	Elevated upper body position 45°	15	33.53 (5.7)	0/15	–	–	–	–
				Placebo	Non-elevated position	15	33.53 (5.7)	0/15		–	–	–
Eijsvogel (22)	Patients werediagnosed with OSAS If they met the following criteria as defined by the American Academy of Sleep Medicine (AASM):complaints of excessive daytime sleepiness (naps duringday/evening) or ≥ 2 of the following that were not better explained by other factors: choking or gasping during sleep, recurrent awakenings from sleep, refreshing sleep, daytimefatigue, and/or impaired concentration, in combination withan AHI ≥ 5.	Parallel RCT	The Netherlands	SPT	NightBalance, a small device placed in a pocket of a neoprene strap attached to the patients’ chest.	29	50.1 (10.6)	23/6	1	–	–	–
				Placebo	TBT: three inflated airbags positioned on the back with an elastic band around the chest	26	50.7 (12.2)	22/4		–	–	–
Stavrou (39)	Patientsreferred for potential sleep disordered breathing following apolysomnography study, an apnea-hypopnea index (AHI) of ≥ 5 events/h with LP, age between 20 and 80 years, BMI < 40kg/m^2^, waist to hip ratio < 1, and neck circumferences < 40 cm	Preliminary RCT	Greece	LP	3 h with LP and 3 h with OP	20	53.8 (12.5)	16/4	3	–	–	–
				Placebo	3 h with LP and 3 h with MFP	12	52 (6.3)	8/4		–	–	–
**SPT Versus OAT**												
Huang (30)	Patients with POSA, diagnosed by standard PSG	Parallel RCT	China	SPT	VVFLY SnoreCircle	20	39.2 (10.92)	17/3	6	89.47%		10.53%
				OAT	Somnofit	20	41.55 (11.79)	18/2		90.00%		10%
				SOT	VVFLY SnoreCircle + Somnofit	20	40.75 (10.51)	16/4		90.00%		10%
de Ruiter (20)	Patients with mild to moderate POSA (AHI 5–30/h)	Parallel RCT	The Netherlands	SPT	NightBalance worn across the chest using a neoprene strap	48	47.3 (10.1)	34/14	12	100%		–
				OAT	SomnoDent flex, a custom-made duo-bloc device, where the OA was set at 60%	51	49.2 (10.2)	36/15		100%		–
Suzuki (40)	Patients suspected to have OSA	Parallel RCT	Japan	PTD	NightShift, choker-type, electronic device worn around the neck	80	45.6 (11.4)	54/26	2	–	–	–
				OAT	Self-cured acrylic resin	80	47.5 (11.4)	62/18		–	–	–
Benoist (28)	Patients with mild-to-moderate POSA (AHI insupine position at least twice as high as compared with the AHI innon-supine position, with 10e90% of TST in thesupine position, and aged > 18 years)	Multicenter, parallel RCT	The Netherlands	SPT	NightBalance B.V.TM worn across the chest using a neoprene strap	48	47.3 (10.1)	34/14	3	100%		–
				OAT	SomnDent flexTM, a custom-made titratable device worn intraorally that had a soft inner liner to maintain retention and support comfort	51	49.2 (10.2)	36/15		100%		–
Dieltjens (21)	Patients with residual, supine-dependent, moderate to severe OSA (AHI > 20 events/h)	Parallel RCT	Belgium	SPT	NightBalanceTM, a chest-worn SPT placed at the level of the sternum. It monitors body positions and vibrates when lying in the supine position.	20	52 (11)	11/9	6	–	100%	
				MAD	Respident Butterfly, a custom-made titratable MAD - oral appliance	20	52 (11)	11/9		–	100%	
				SPT + MAD	NightBalance + Respident Butterfly	20	52 (11)	11/9		–	100%	
**SPT Versus CPAP**												
Berry (29)	Patients with ePOSA (AHI ≥ 15 events/h and nsAHI < 10events/h) or (AHI > 10 and < 15 events/h with daytime sleepiness and nsAH < 5 events/h)	Cross-over RCT	United States	SPT	NightBalance, a rechargeable battery-operated device worn around the chest in an elasticized torso band which contains a digital accelerometer. A vibration is given when the patient turns to the supine position	58	50.8 (12.6)	34/24	1.5	14 (24.13%)	44 (75.87%)	
				CPAP	Dreamstation Auto, with a pressure range setting of 4–20 cm H_2_O and a mask as tolerated. This device comes with supplementary Wisp (nasal), Nuance (nasal pillows), and Amara View (full face).	59	51.57 (12.7)	36/23		11 (18.64%)	45 (75.87%)	
Jokic (32)	Patients with OSA	Cross-over RCT	Canada	PT	Backpack with a soft ball inside, positioned to prevent the patient from sleeping supine. It was made out of semirigid synthetic foam	13	51 (9)	–	0.5	–	–	–
				CPAP	For 2 weeks	13	51 (9)	–		–	–	–
Mok (35)	POSA patients with ESS > = 10	Cross-over RCT	Singapore	PT	Night Shift positional device worn at the back of the neck using a latex-free silicone rubber strap.	20	–	–	2	–	–	–
				CPAP	Airsense 10 (Resmed) in automated mode	21	–	–		–	–	–
Permut (36)	POSA patients (AHI of at least five eventsper hour with symptoms of excessive daytime sleepiness or anAHI of at least 15 events per hour with a 50% decrease in theAHI when the patient was sleeping in the nonsupine position, as compared with in the supine position)	Multicenter cross-over RCT	United States	PT	Zzoma Positional Sleeper made of lightweight semirigid synthetic foam	38	49 (12)	25/13	3	29 (76.3%)	9 (23.7%)	–
				CPAP	CPAP at 5 cm H_2_O titrated upward in 2 cm H_2_O increment	38						–
Skinner (38)	POSA patients with AHI 10–60/h	Cross-over RCT	New Zealand	SHEP	Embletta PDFTM, a standardized foam wedge, the top surface of which was angled at 60°to the horizontal	14	54 (10)	12/2	1	100%		–
				nCPAP	Using an autotitrating machine (Autoset T)	14	54 (10)	12/2		100%		–
Skinner (37)	Patients with moderate to severe POSA (mean AHI of 22.7)	Cross-over RCT	New Zealand	TASB	It contains two-equal lengths of cotton stockinette-covered 6 mm foam rubber	10	55.9 (9.8)	–	1	–	100%	
				nCPAP	Using an autotitrating machine (Autoset T)	10	55.9 (9.8)	–		–	100%	

YOP, year of publication; No, number of patients; RCT, randomized controlled trial; FU, follow-up; M/F, male/female; Mod, moderate; Sev, severe; OSA, obstructive sleep apnea; AHI, apnea-hypopnea index; OAT, oral appliance therapy; SPT, sleep positional therapy; APAP, auto-adjusting positive airway pressure; MAD, mandibular advancement device; TBT, tennis ball technique; SPMD, sleep position modification device; PT, positional therapy; SHEP, shoulder-head elevation pillow; TASB, thoracic anti-supine band; MFP, memory foam pillow; LP, laboratory pillow; op, own pillow; PTD, positional therapy device.

Examined SPT interventions included Somnibel (*n* = 1), NightBalance (*n* = 5), Buzz-POD (*n* = 1), Respident Butterfly (*n* = 1), SPMD (Sleep Position Modification Device) (*n* = 1), backpack (*n* = 1), head elevation technique (*n* = 2), Night Shift (*n* = 2), Zzoma (*n* = 1), Embletta SHEP (shoulder-head elevation device) (*n* = 1), TASB (thoracic anti-supine band) (*n* = 1), and laboratory pillows (*n* = 2). A full description of allocated interventions, patients’ age and gender, and follow-up duration can be found in Table 1.

### 3.3 Risk of bias summary

**FIGURE 2:**
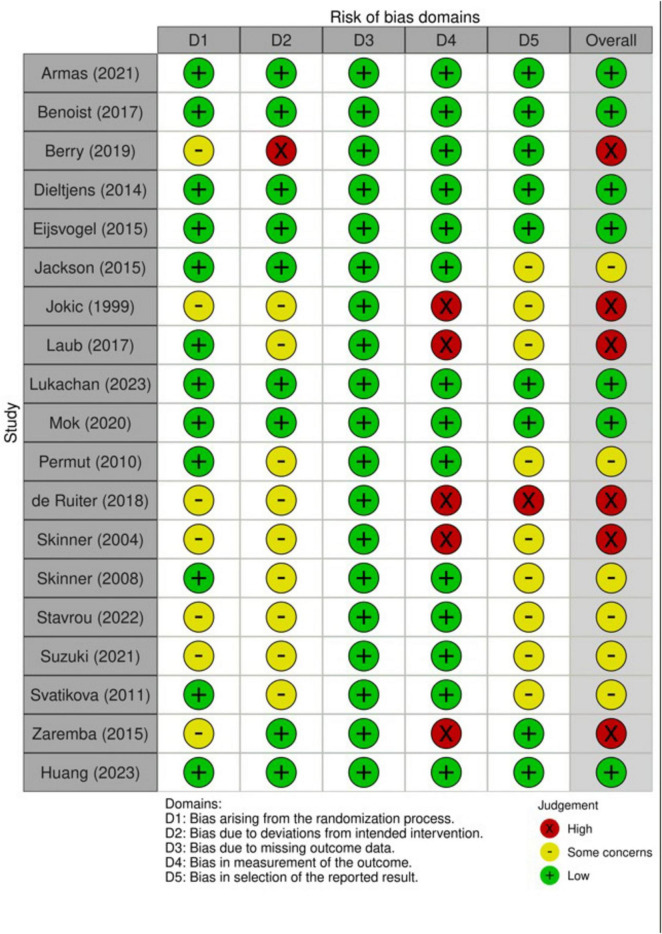
An illustration of the risk of bias summary of included RCTs based on Cochrane’s revised RoB2 tool. RCT, randomized controlled trial.

The results of the RoB assessment are provided in Figure 2. Overall, seven RCTs had low risk of bias, six RCT had high risk of bias, while the remaining six RCTs had some concerns. The main concerns were attributed to the lack of a study protocol to examine reporting bias (*n* = 10), deviations from intended interventions (lack of reporting, *n* = 10), and improper description of the randomization process (*n* = 7).

### 3.4 Primary endpoint(s)

#### 3.4.1 Total AHI score

• Sleep positional therapy vs. Placebo: No difference was noted between groups (five studies, MD = −1.66; 95% CI: −3.76, 0.4). The degree of heterogeneity was insignificant (I^2^ = 51.12%, *p* = 0.09). The severity of OSA, type of SPT, and follow-up period did not affect the observed results (Supplementary Figure 1).

Sleep positional therapy vs. OAT: No difference was noted between groups (five studies, MD = 0.88, 95% CI: −0.57, 2.33). No heterogeneity was observed (I^2^ = 0%, *p* = 0.43). This effect was consistent across different follow-up points, SPT types, and OSA severity groups (Supplementary Figure 2).

**FIGURE 3:**
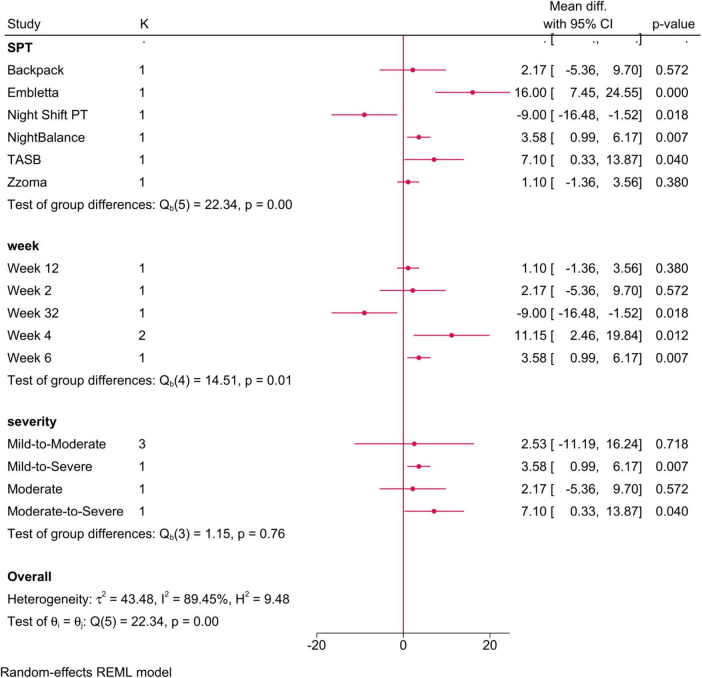
Forest plot showing the difference in overall AHI score between sleep positional therapy (SPT) and CPAP, stratified by SPT type, follow-up period, and OSA severity. OSA, obstructive sleep apnea; CPAP, continuous positive airway pressure; AHI, apnea-hypopnea index.

• Sleep positional therapy vs. CPAP: No difference was observed between both groups (MD = 3.28; 95% CI: −2.56, 9.12, I^2^ = 89.45%, *p* = 0.001). However, after removing the study of Mok et al. (35) in the sensitivity analysis, SPT exhibited a greater change in AHI score (MD = 5; 95% CI: 0.66, 9.35) (Supplementary Figure 3). Both SPT type and follow-up period significantly modified the observed effect (Figure 3), with only Embletta (positive change) and Night Shift PT (negative change) showing a significant change.

#### 3.4.2 AHI score in supine position

**FIGURE 4:**
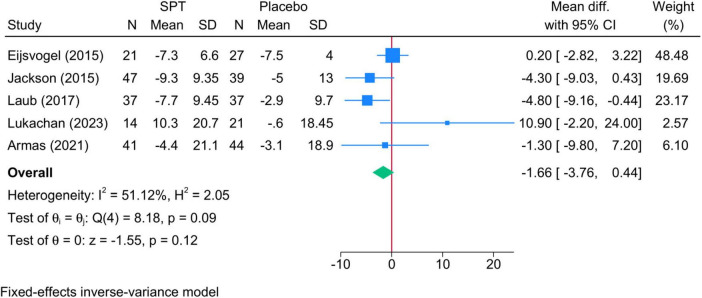
Forest plot showing the difference in AHI score in supine position between SPT and placebo. AHI, apnea-hypopnea index; SPT, sleep positional therapy.

• Sleep positional therapy vs. Placebo: SPT was associated with a greater reduction in AHI in supine position compared to placebo (four studies, MD = −7.46; 95% CI: −11.42, −3.49) (Figure 4). The degree of heterogeneity was considerable (I^2^ = 98.60%, *p* = 0.001); however, no change was observed in the sensitivity analysis.
• Sleep positional therapy vs. OAT: No difference was noted between groups (four studies, MD = 6.96; 95% CI: −4.90, 18.83). Heterogeneity was considerable (I^2^ = 99.91%, *p* = 0.001); and the sensitivity analysis showed a greater change in the SPT group after excluding the study of de Ruiter et al. (20) (MD = 11.64; 95% CI: 0.94, 22.34).
• Sleep positional therapy vs. CPAP: No difference was observed between both groups (four studies, MD = 7.33; 95% CI: −7.63, 22.30, I^2^ = 99.86%, *p* = 0.001). However, the sensitivity analysis revealed a greater increase in favor of SPT after removing the study of Mok et al. (35) (MD = 13.97; 95% CI: 3.54, 24.40) (Supplementary Figure 4).

#### 3.4.3 AHI in non-supine position

• Sleep positional therapy vs. Placebo: No difference was noted between groups (four studies, MD = −0.66; 95% CI: −3.43, 2.1). The degree of heterogeneity was considerable (I^2^ = 97.54%, *p* = 0.001); however, no change was observed in the sensitivity analysis.
• Sleep positional therapy vs. OAT: No difference was noted between groups (four studies, MD = −0.91; 95% CI: −6.25,4.43). Heterogeneity was considerable (I^2^ = 99.74%, *p* = 0.001); and the sensitivity analysis revealed a greater increase in favor of SPT after removing the study of Dieltjens et al. (21) (MD = 2.20; 95% CI: 1.52, 2.88) (Supplementary Figure 5).
• Sleep positional therapy vs. CPAP: No difference was observed between both groups (four studies, MD = 1.74; 95% CI: −6.60, 10.08, I^2^ = 99.67%, *p* = 0.001). However, the sensitivity analysis revealed a greater increase in AHI score after removing the study of Mok et al. (35) (MD = 5.87; 95% CI: 3.91, 7.84) (Supplementary Figure 6).

### 3.5 Secondary endpoints: sleep indices

#### 3.5.1 TST % (overall)

• Sleep positional therapy vs. Placebo: No difference was observed between groups (three studies, MD = −1.48; 95% CI: −17.79, 14.83). Heterogeneity was considerable (I^2^ = 97.62%, *p* = 0.001); however, the sensitivity analysis was unremarkable.
• Sleep positional therapy vs. OAT: Insufficient evidence.
• Sleep positional therapy vs. CPAP: No difference was observed between both groups (four studies, MD = −3.31; 95% CI: −15.02, 8.41; I^2^ = 90.98%, *p* = 0.001). The sensitivity analysis revealed no change.

#### 3.5.2 TST % in supine position

• Sleep positional therapy vs. Placebo: No difference was observed between groups (three studies, MD = −2.47; 95% CI: −7.40, 2.47). Heterogeneity was insignificant (I^2^ = 43.35%, *p* = 0.17).
• Sleep positional therapy vs. OAT: No difference was observed between groups (five studies, MD = −21.68; 95% CI: −60.09, 16.73, I^2^ = 98.84%, *p* = 0.001). However, the sensitivity analysis revealed a significantly greater reduction in TST is supine position in favor of SPT (MD = −39.63; 95% CI: −60.71, −18.56) after removing the study of Dieltjens et al. (21) (Supplementary Figure 7). The SPT type, follow-up period, and OSA severity were significant effect modifiers (Supplementary Figure 8). For instance, SPT exhibited a lower change in TST in supine position in patients with mild/moderate OSA, while it exhibited a greater increase in those with moderate/severe disease.
• Sleep positional therapy vs. CPAP: No difference was observed between both groups (three studies, MD = −6.14; 95% CI: −61.42, 49.15, I^2^ = 99.04%, *p* = 0.001). However, the sensitivity analysis revealed a significantly greater reduction in favor of SPT (MD = −35.65; 95% CI: −45.08, −26.23) after removing the study of Permut et al. (36) (Supplementary Figure 9).

#### 3.5.3 TST % in N1 sleep

• Sleep positional therapy vs. Placebo: No difference was observed between groups (two studies, MD = −1.07; 95% CI: −2.30, 0.17). Heterogeneity was insignificant (I^2^ = 69.74%, *p* = 0.07).
• Sleep positional therapy vs. OAT: No difference was noted between groups (two studies, MD = −0.67; 95% CI: −1.52, 0.19, I^2^ = 0%, *p* = 0.54).
• Sleep positional therapy vs. CPAP: Insufficient evidence.

#### 3.5.4 TST % in N2 sleep

**FIGURE 5:**
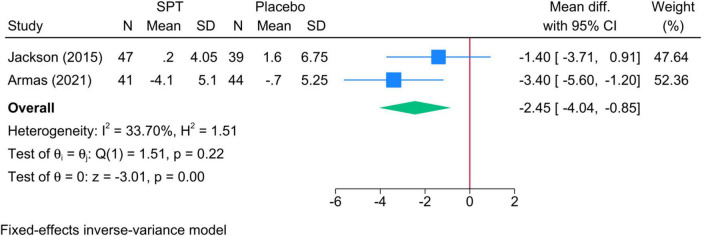
Forest plot showing the difference in TST in N2 sleep stage between SPT and placebo. TST, total sleep time; SPT, sleep positional therapy.

• Sleep positional therapy vs. Placebo: The mean reduction in TST during N2 was significantly greater in SPT than placebo (two studies, MD = −2.45; 95% CI: −4.04, −0.85) (Figure 5). Heterogeneity was insignificant (I^2^ = 69.74%, *p* = 0.07).
• Sleep positional therapy vs. OAT: SPT exhibited a greater mean reduction than OAT (two studies, MD = −2.79; 95% CI: −4.53, −1.04, I^2^ = 34.84%, *p* = 0.22).
• Sleep positional therapy vs. CPAP: Insufficient evidence.

#### 3.5.5 TST % in N3 sleep

**FIGURE 6:**
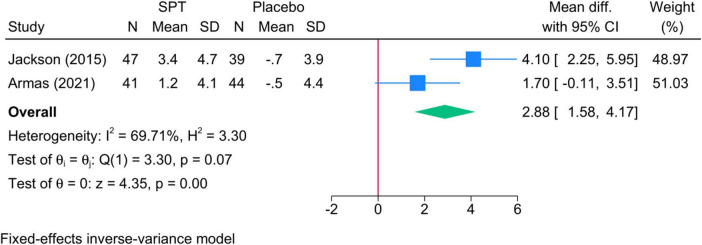
Forest plot showing the difference in TST in N3 sleep stage between SPT and placebo. TST, total sleep time; SPT, sleep positional therapy.

• Sleep positional therapy vs. Placebo: The mean change in TST during N3 was significantly greater in SPT than placebo (two studies, MD = 2.88; 95% CI: 1.58, 4.17) (Figure 6). Heterogeneity was insignificant (I^2^ = 69.71%, *p* = 0.07).
• Sleep positional therapy vs. OAT: SPT exhibited a greater increase in meant TST during N3 compared to OAT (two studies, MD = 3.64; 95% CI: 1.20, 6.09, I^2^ = 79.67%, *p* = 0.03).
• Sleep positional therapy vs. CPAP: Insufficient evidence.

#### 3.5.6 TST % in REM

• Sleep positional therapy vs. Placebo: No difference was observed between groups (two studies, MD = −1.70; 95% CI: −3.47, 0.06), but heterogeneity was considerable (I^2^ = 95.74%, *p* = 0.001).
• Sleep positional therapy vs. OAT: Insufficient evidence.
• Sleep positional therapy vs. CPAP: Insufficient evidence.

#### 3.5.7 CT90 (% TST with SaO_2_ < 90%)

• Sleep positional therapy vs. Placebo: No difference was observed between groups (three studies, MD = 4.07; 95% CI: −6.93, 15.06). Although heterogeneity was considerable (I^2^ = 93.20%, *p* = 0.001), the sensitivity analysis was unremarkable.
• Sleep positional therapy vs. OAT: Insufficient evidence.
• Sleep positional therapy vs. CPAP: No difference was observed between both groups (two studies, MD = 1.62; 95% CI: −1.47, 4.72, I^2^ = 98.31%, *p* = 0.001).

### 3.6 Secondary endpoints: respiratory indices

#### 3.6.1 Sleep efficiency

• Sleep positional therapy vs. Placebo: No difference was noted between both groups (three studies, MD = −3.59; 95% CI: −7.22, 0.05). No heterogeneity was noted (I^2^ = 0%, *p* = 0.38).
• Sleep positional therapy vs. OAT: No difference was observed between group (five studies, MD = 0.43; 95% CI: −2.41, 3.27, I^2^ = 0%, *p* = 0.76). This observation was consistent in different SPT types, follow-up periods, and OSA severity groups (Supplementary Figure 10).
• Sleep positional therapy vs. CPAP: No difference was observed between both groups (three studies, MD = 1.57; 95% CI: −3.74, 6.88, I^2^ = 64.33%, *p* = 0.05). However, the sensitivity analysis showed a greater increase in sleep efficiency in favor of SPT after removing the study of Permut et al. (36) (MD = 4.23; 95% CI: 1.07, 7.38) (Supplementary Figure 11).

#### 3.6.2 Arousal index

• Sleep positional therapy vs. Placebo: No difference was observed between groups (three studies, MD = −1.74; 95% CI: −5.35, 1.87). Although heterogeneity was considerable (I^2^ = 99.68%, *p* = 0.001), the sensitivity analysis was unremarkable.
• Sleep positional therapy vs. OAT: The mean reduction in arousal index was significantly greater in the SPT group (MD = −7.11; 95% CI: −10.52, −3.71) (Supplementary Figure 12). Heterogeneity was considerable (I^2^ = 99.13%, *p* = 0.001); however, the sensitivity analysis was unremarkable.
• Sleep positional therapy vs. CPAP: SPT exhibited a greater reduction in arousal indexed compared to CPAP (three studies, MD = −3.22; 95% CI: −4.82, −1.61, I^2^ = 98.51%, *p* = 0.001). The sensitivity analysis was unremarkable.

#### 3.6.3 Minimum SaO_2_

• Sleep positional therapy vs. Placebo: No difference was observed between groups (three studies, MD = −2.59; 95% CI: −6.85, 1.66). Although heterogeneity was considerable (I^2^ = 99.09%, *p* = 0.001), the sensitivity analysis was unremarkable.
• Sleep positional therapy vs. OAT: No change was observed between groups (two studies, MD = −0.28; 95% CI: −1.67, 1.11, I^2^ = 10.43%, *p* = 0.29).
• Sleep positional therapy vs. CPAP: No difference was observed between both groups (two studies, MD = 0.64; 95% CI: −5.87, 7.15; I^2^ = 98.36%, *p* = 0.001).

#### 3.6.4 Mean SaO_2_

• Sleep positional therapy vs. Placebo: No difference was observed between groups (three studies, MD = −0.58; 95% CI: −1.27, 0.11). Heterogeneity was moderate (I^2^ = 70.26%, *p* = 0.05), and the sensitivity analysis revealed a significantly greater reduction in mean SaO_2_ in favor of SPT after removing the study of Laub et al. (33) (MD = −0.84; 95% CI: −1.33, −0.35) (Supplementary Figure 13).
• Sleep positional therapy vs. OAT: No change was observed between groups (two studies, MD = 0.18; 95% CI: −0.80, 1.16, I^2^ = 0%, *p* = 0.35).
• Sleep positional therapy vs. CPAP: SPT resulted in greater reduction than CPAP (two studies, MD = −1.64; 95% CI: −2.33, −0.96, I^2^ = 92.58%, *p* = 0.001).

#### 3.6.5 ODI score

• Sleep positional therapy vs. Placebo: The mean change in ODI score was significantly higher in the SPT group (three studies, MD = 2.20; 95% CI: 0.68, 3.72). Heterogeneity was moderate (I^2^ = 70.17%, *p* = 0.04), and the sensitivity analysis revealed no difference between both groups after removing the study of Eijsvogel et al. (22) (MD = 1.92; 95% CI: −1.00, 4.83) (Supplementary Figure 14).
• Sleep positional therapy vs. OAT: No difference was observed between groups (four studies, MD = 0.01; 95% CI: −1.06, 1.09, I^2^ = 94.67%, *p* = 0.00). The sensitivity analysis was unremarkable.
• Sleep positional therapy vs. CPAP: No difference was observed between both groups (two studies, MD = −1.42; 95% CI: −8.61, 5.78; I^2^ = 99.57, *p* = 0.001).

### 3.7 Secondary endpoints: clinical parameters

#### 3.7.1 FOSQ score

• Sleep positional therapy vs. Placebo: Insufficient evidence.
• SPT vs. OAT: No difference was observed between groups (two studies, MD = 1.06; 95% CI: −0.21, 2.33, I^2^ = 97.65%, *p* = 0.001).
• Sleep positional therapy vs. CPAP: No difference was observed between both groups (four studies, MD = −0.10; 95% CI: −0.56, 0.35, I^2^ = 93.58%, *p* = 0.001). However, after removing the study of Mok et al. (35), SPT exhibited a greater reduction in FOSQ than CPAP (MD = −0.32; 95% CI: −0.43, −0.20) (Supplementary Figure 15).

#### 3.7.2 ESS score

• Sleep positional therapy vs. Placebo: No difference was observed between groups (four studies, MD = −0.56, −1.73, 0.60). Heterogeneity was considerable (I^2^ = 97.45%, *p* = 0.001), and the sensitivity analysis revealed a significantly greater reduction in ES score in favor of SPT after removing the study of Eijsvogel et al. (22) (MD = −1.04; 95% CI: −2.00, −0.08) (Supplementary Figure 16).
• Sleep positional therapy vs. OAT: No difference was observed between groups (three studies, MD = 0.89; 95% CI: −0.18, 1.96). Heterogeneity was considerable (I^2^ = 91.40%, *p* = 0.001); however, the sensitivity analysis revealed a greater increase in favor of SPT (MD = 1.58; 95% CI: 1.0, 2.10) after removing the study of de Ruiter et al. (20) (Supplementary Figure 17).
• Sleep positional therapy vs. CPAP: SPT exhibited a greater increase compared to CPAP (MD = 0.92; 95% CI: 0.66, 1.19, I^2^ = 0%, *p* = 0.55) (Supplementary Figure 18).

#### 3.7.3 Treatment response

• Sleep positional therapy vs. Placebo: No difference in the odds of response was observed (two studies, OR = 2.03; 95% CI: 0.67, 6.13, I^2^ = 49.31%, *p* = 0.15).
• Sleep positional therapy vs. OAT: Insufficient evidence.
• Sleep positional therapy vs. CPAP: Insufficient evidence.

#### 3.7.4 Complications

• Sleep positional therapy vs. Placebo: Insufficient evidence.
• Sleep positional therapy vs. OAT: The risk of complications was significantly lower in the SPT group (two studies, OR = 0.54; 95% CI: 0.31, 0.95) (Supplementary Figure 19). No heterogeneity was observed (I^2^ = 4.80%, *p* = 0.31).
• Sleep positional therapy vs. CPAP: SPT exhibited a lower risk of complication than CPAP (OR = 0.29; 95% CI: 0.12, 0.72, I^2^ = 0%, *p* = 0.36).

#### 3.7.5 Persistent apnea

• Sleep positional therapy vs. Placebo: Insufficient evidence.
• Sleep positional therapy vs. OAT: No difference in the risk was observed between groups (two studies, OR = 1.50; 95% CI: 0.30,7.44). No heterogeneity was observed (I^2^ = 0%, *p* = 0.63).
• Sleep positional therapy vs. CPAP: Insufficient evidence.

#### 3.7.6 Persistent snoring

• Sleep positional therapy vs. Placebo: Insufficient evidence.
• Sleep positional therapy vs. OAT: No difference in the risk was observed between groups (OR = 0.95; 95% CI: 0.49, 1.81, I^2^ = 0%, *p* = 0.97).
• Sleep positional therapy vs. CPAP: Insufficient evidence.

#### 3.7.7 Persistent tiredness

• Sleep positional therapy vs. Placebo: Insufficient evidence.
• Sleep positional therapy vs. OAT: No difference in the risk was observed between groups (OR = 0.61; 95% CI: 0.30, 1.24, I^2^ = 0%, *p* = 0.60).
• Sleep positional therapy vs. CPAP: Insufficient evidence.

## 4 Discussion

### 4.1 Principal findings

This systematic review and meta-analysis compared the efficacy and safety of SPT with placebo, OAT, and CPAP in patients with OSA. While SPT offers a less invasive alternative to CPAP, our findings suggest mixed outcomes, particularly when considering the clinical significance of improvements in key metrics such as AHI, sleep architecture, and safety.

### 4.2 Effectiveness of SPT vs. placebo

Our analysis indicates that SPT shows a greater reduction in AHI during the supine position compared to placebo, with a mean difference of −7.46 (95% CI: −11.42, −3.49), albeit with considerable heterogeneity. This outcome aligns with previous findings that highlight the efficacy of SPT in reducing AHI in patients with positional OSA, where the supine position exacerbates airway collapse (42, 43). However, no significant difference was observed in the overall AHI, non-supine AHI, or TST, suggesting that the benefit of SPT may be limited to patients with supine-related OSA. Notably, no changes were observed in other secondary sleep parameters, such as N1 and REM sleep. These results imply that SPT’s primary value may lie in its ability to modulate positional apnea, without a broader impact on overall sleep quality.

The lack of a significant effect on total sleep time and sleep efficiency in SPT-treated patients compared to placebo raises important questions about its role in improving sleep architecture. Sleep quality, including the percentage of time spent in deeper sleep stages such as N3, is crucial for restorative sleep and cognitive functioning. In our study, SPT did not significantly improve these parameters, which suggests that while SPT is effective in reducing supine-related apneas, it does not provide the same holistic improvements in sleep structure as CPAP or other more aggressive therapies.

### 4.3 SPT vs. OAT

When compared to OAT, SPT did not demonstrate significant advantages in terms of reducing overall AHI or AHI in the supine position, although sensitivity analyses indicated a trend favoring SPT after the exclusion of certain studies [e.g., de Ruiter et al. (20)]. OAT has been shown to improve AHI by repositioning the mandible, thereby preventing airway collapse, particularly in mild to moderate OSA. The lack of significant difference between SPT and OAT suggests that both modalities may be appropriate for patients who cannot tolerate CPAP, offering similar efficacy in reducing apnea events.

However, in the comparison of other sleep-related indices, SPT showed greater improvements in arousal index than OAT, with a significant mean reduction of −7.11 (95% CI: −10.52, −3.71). This suggests that SPT may offer better sleep continuity by reducing night-time awakenings compared to OAT. This result is clinically relevant, as frequent arousals are associated with fragmented sleep, reduced daytime alertness, and increased cardiovascular risk (44, 45). Nevertheless, given the considerable heterogeneity in these results, further research is needed to validate the observed advantages of SPT over OAT in this domain.

Several studies have also compared the efficacy of SPT to OAT. A recent meta-analysis by Mohamed et al. (14) demonstrated that while both therapies are effective in addressing positional OSA, OAT was associated with greater improvements in non-supine AHI and ESS scores?. Our findings align with this, as we observed no significant differences in total AHI between SPT and OAT, but noted that OAT was superior in improving daytime sleepiness and non-supine AHI. Nevertheless, SPT outperformed OAT in terms of reducing supine sleep time, which highlights its value for patients with strong positional OSA tendencies.

### 4.4 SPT vs. CPAP

Our analysis showed that CPAP remains superior to SPT in reducing overall AHI, particularly in non-supine positions. While SPT did not show significant differences compared to CPAP in most key outcomes, sensitivity analyses revealed a greater increase in AHI and a decrease in SaO_2_ in favor of SPT when the study of Mok et al. (35) was excluded. These findings reinforce CPAP’s status as the gold standard for OSA treatment (46), particularly in its ability to consistently lower AHI across various positional settings and improve oxygenation.

However, the use of SPT did demonstrate some advantages over CPAP in terms of safety and comfort, as evidenced by a significantly lower risk of device-related complications. The pooled risk ratio for complications was lower in the SPT group compared to CPAP (OR = 0.29; 95% CI: 0.12, 0.72). The lower rate of complications, such as dry mouth (28), discomfort (9), and other adverse effects, highlights the value of SPT for patients who are intolerant to CPAP. Despite its inferiority in AHI reduction, the relative comfort and safety of SPT suggest it could be a viable option for patients with mild to moderate OSA who cannot adhere to CPAP therapy.

Previous meta-analyses have documented the efficacy of CPAP over SPT for reducing AHI and improving oxygen saturation levels. Ha et al. (10) found that while SPT reduces AHI in the supine position, CPAP remains superior in terms of overall efficacy and oxygenation?. Our findings are consistent with these results; although we observed improvements in supine AHI with SPT compared to placebo, CPAP demonstrated greater improvements in overall AHI and oxygenation, especially in patients with moderate to severe OSA. However, our analysis highlights that SPT still offers a viable alternative for patients who cannot tolerate CPAP, as it significantly reduces the percentage of time spent in the supine position, which may benefit those with positional OSA.

### 4.5 Impact on sleep architecture and other secondary outcomes

In our evaluation of sleep architecture, SPT demonstrated inconsistent results. While it was associated with greater improvements in N3 sleep when compared to placebo and OAT, no significant difference was observed for REM sleep or N1 sleep across all comparisons. This finding is particularly relevant because deeper sleep stages, such as N3, are critical for physical recovery and overall well-being (47). However, the lack of improvement in REM sleep raises concerns, as REM sleep is essential for cognitive functioning and emotional regulation. These mixed outcomes suggest that while SPT may confer some benefits in sleep architecture, its effects are not uniform across all sleep stages, and further research is needed to understand its long-term implications on sleep quality and patient outcomes.

### 4.6 Oxygen saturation and respiratory indices

No significant differences were found between SPT and placebo, OAT, or CPAP in mean SaO_2_ or minimum SaO_2_. This lack of improvement in oxygen saturation parameters might be due to the nature of SPT, which primarily targets positional airway collapse without directly addressing hypoxic events (32). In contrast, CPAP has consistently been shown to improve oxygenation, further emphasizing its superiority in managing OSA-related hypoxemia. Interestingly, the ODI score was significantly higher in the SPT group compared to placebo, suggesting a potential worsening of intermittent hypoxia in some patients. Although this result requires further investigation, it may indicate that SPT is not suitable for all OSA phenotypes, particularly those with severe desaturations.

The role of newer generation SPT devices, such as those employing vibrational alarms, has been investigated in several recent reviews. Ravesloot et al. (13) concluded that these devices are highly effective in reducing AHI and percentage of supine sleep, with good short-term compliance, though long-term adherence remains an area of concern?. In our analysis, the vibrational SPT devices like the NightBalance and Zzoma devices demonstrated greater reductions in supine AHI compared to older methods, such as tennis ball techniques. However, compliance remains an important consideration. The short-term nature of most studies included in our review limits our ability to draw strong conclusions about long-term adherence, a critical factor for the sustained success of any therapeutic modality.

The observed heterogeneity across studies was attributed to variations in study populations (e.g., severity of OSA, comorbid conditions), intervention protocols (e.g., differences in CPAP settings, oral appliance designs, or sleep positional therapy approaches), and outcome measures. Subgroup analyses based on OSA severity (Supplementary Figures 1, 2, 8, 10) and sensitivity analyses were conducted to explore potential sources of heterogeneity. These findings suggest that differences in baseline characteristics and methodological approaches among studies contribute significantly to the observed variability.

### 4.7 Study limitations, clinical implications, and future research

This study has several limitations that warrant consideration. First, there was substantial heterogeneity in many of the analyses, particularly when comparing SPT to placebo and OAT, which may have influenced the robustness of the results. The variability in study designs, types of SPT devices, and follow-up durations across included trials contributed to this heterogeneity, limiting the generalizability of the findings. Second, while sensitivity analyses were performed to mitigate the impact of outliers, the exclusion of individual studies significantly altered some results, suggesting potential biases in the data. Third, the inclusion of a placebo group in which patients remained in a supine position may have artificially inflated the efficacy of SPT, as the supine position is known to worsen OSA. Moreover, trial sequential analysis (TSA) was not performed in this meta-analysis due to the limited number of included studies and the lack of key parameters (e.g., anticipated effect size, event rates, and variance) required for robust TSA. Future studies with larger datasets may enable the application of TSA to further validate the findings. Despite the limited number of included studies, the potential for publication bias cannot be ruled out. The absence of small or negative studies in the analysis may result in an overestimation of the effect sizes. Although formal tests for publication bias (e.g., Egger’s test) were not feasible due to insufficient study numbers, visual inspection of funnel plots (where applicable) did not indicate substantial asymmetry. Finally, the small sample sizes in some subgroup analyses and the limited number of high-quality trials available for certain outcomes (e.g., TST in N1, N2, and REM) reduce the power to draw definitive conclusions. Further large-scale randomized controlled trials are needed to validate these findings and provide a more comprehensive understanding of SPT’s role in OSA management.

The findings of this meta-analysis provide valuable insights for clinical practice. The superior efficacy of CPAP in improving key clinical outcomes such as the apnea-hypopnea index and ESS scores underscores its role as the gold standard treatment for OSA. However, the comparable benefits of OAT and SPT in specific secondary outcomes highlight their potential as alternative options, particularly for patients who are intolerant to CPAP or have positional OSA. These results support a patient-centered approach, where the choice of therapy is guided by individual preferences, severity of OSA, and specific clinical characteristics. Moreover, the association between OSA treatment and reductions in oxidative stress and systemic inflammation further emphasizes the importance of timely intervention to mitigate the risk of comorbidities such as cardiovascular and metabolic disorders. Integrating these findings into routine practice may enhance patient outcomes and reduce the long-term burden of OSA-related complications.

Future research should focus on addressing several important gaps identified in this meta-analysis. Larger, multi-center randomized controlled trials are essential to validate findings, particularly for interventions such as oral appliance therapy and sleep positional therapy, where evidence remains limited. Investigating the long-term effects of OSA treatments on oxidative stress, systemic inflammation, and related comorbidities will provide a more comprehensive understanding of therapeutic impacts. Additionally, exploring the role of novel biomarkers and advanced diagnostic tools may facilitate personalized treatment strategies, optimizing outcomes for diverse patient populations. Economic evaluations of different treatment modalities will also be valuable to inform healthcare policies and ensure equitable access to effective care. By addressing these gaps, future studies can strengthen the evidence base and enhance clinical management of OSA.

SPT provides a safe and viable alternative to CPAP and OAT for managing OSA, particularly in patients with positional OSA or those who are intolerant to CPAP. However, it remains inferior to CPAP in terms of reducing overall AHI and improving oxygenation, and its impact on sleep architecture is mixed. Clinicians should consider patient preferences, OSA severity, and positional dependency when recommending SPT as a treatment option. Further research is required to optimize its use and to better understand the patient populations that would benefit most from this therapy.

## Data Availability

The raw data supporting the conclusions of this article will be made available by the authors, without undue reservation.
