# Supplementary material for: Comparative efficacy of sleep positional therapy, oral appliance therapy, and CPAP in obstructive sleep apnea: a meta-analysis of mean changes in key outcomes
Source: Front Med (Lausanne). 2025 Feb 3;12:1517274. doi: 10.3389/fmed.2025.1517274 (PMC11830591; doi:10.3389/fmed.2025.1517274)
Supplement: Supplementary file 1 [file Table_1.docx]

**Supplementary Table 1.** The detailed search query employed in the literature search

| Database | No | Search Query | Results |
| --- | --- | --- | --- |
| PubMed [Date of search: 04/06/2024] | | | |
|  | #1 | “obstructive sleep apnea”[tiab] OR “Sleep Apnea Syndrome”[tiab] OR “Sleep Apnea Hypopnea”[tiab] OR “obstructive sleep apnoea”[tiab] OR “positional sleep apnea”[tiab] OR OSAS[tiab] OR OSAHS[tiab] OR OSA[tiab] OR POSA[tiab] OR "Sleep Apnea, Obstructive"[Mesh] | 47466 |
|  | #2 | Random*[tiab] OR "Randomized Controlled Trial" [Publication Type] | 1655749 |
|  | #3 | “positional therapy”[tiab] OR “positional treatment”[tiab] OR “patient positioning”[tiab] OR “position trainer*”[tiab] OR “tennis ball”[tiab] OR “sleep position*”[tiab] OR pillow*[tiab] OR binder*[tiab] OR backpack*[tiab] OR alarm*[tiab] OR “electrical sensor*”[tiab] OR “electric sensor*”[tiab] OR “positional sleeper”[tiab] OR “positional sleepers”[tiab] OR band*[tiab] OR Sona[tiab] OR posture[tiab] OR position[tiab] OR positional[tiab] OR positioning[tiab] OR supine[tiab] OR vibrati*[tiab] OR “semi-upright”[tiab] OR “side-lying”[tiab] OR “back-lying”[tiab] OR "Patient Positioning"[Mesh] | 1092359 |
|  | #4 | #1 AND #2 AND #3 | 273 |
| Scopus [Date of search: 04/06/2024] | | | |
|  | #1 | TITLE-ABS-KEY (“obstructive sleep apnea”) OR TITLE-ABS-KEY (“Sleep Apnea Syndrome”) OR TITLE-ABS-KEY (“Sleep Apnea Hypopnea”) OR TITLE-ABS-KEY (“obstructive sleep apnoea”) OR TITLE-ABS-KEY (“positional sleep apnea”) OR TITLE-ABS-KEY (OSAS) OR TITLE-ABS-KEY (OSAHS) OR TITLE-ABS-KEY (OSA) OR TITLE-ABS-KEY (POSA) | 116984 |
|  | #2 | TITLE-ABS-KEY (Random*) | 3334869 |
|  | #3 | TITLE-ABS-KEY (“positional therapy”) OR TITLE-ABS-KEY (“positional treatment”) OR TITLE-ABS-KEY (“patient positioning”) OR TITLE-ABS-KEY (“position trainer*”) OR TITLE-ABS-KEY (“tennis ball”) OR TITLE-ABS-KEY (“sleep position*”) OR TITLE-ABS-KEY (pillow*) OR TITLE-ABS-KEY (binder*) OR TITLE-ABS-KEY (backpack*) OR TITLE-ABS-KEY (alarm*) OR TITLE-ABS-KEY (“electrical sensor*”) OR TITLE-ABS-KEY (“electric sensor*”) OR TITLE-ABS-KEY (“positional sleeper”) OR TITLE-ABS-KEY (“positional sleepers”) OR TITLE-ABS-KEY (band*) OR TITLE-ABS-KEY (Sona) OR TITLE-ABS-KEY (posture) OR TITLE-ABS-KEY (position) OR TITLE-ABS-KEY (positional) OR TITLE-ABS-KEY (positioning) OR TITLE-ABS-KEY (supine) OR TITLE-ABS-KEY (vibrati*) OR TITLE-ABS-KEY (“semi-upright”) OR TITLE-ABS-KEY (“side-lying”) OR TITLE-ABS-KEY (“back-lying”) | 5630535 |
|  | #4 | #1 AND #2 AND #3 | 531 |
| Web of Science [Date of search: 04/06/2024] | | | |
|  | #1 | AB=“obstructive sleep apnea” OR AB=“Sleep Apnea Syndrome” OR AB=“Sleep Apnea Hypopnea” OR AB=“obstructive sleep apnoea” OR AB=“positional sleep apnea” OR AB=OSAS OR AB=OSAHS OR AB=OSA OR AB=POSA | 56065 |
|  | #2 | AB=Random* | 2195935 |
|  | #3 | AB=“positional therapy” OR AB=“positional treatment” OR AB=“patient positioning” OR “position trainer*” OR AB=“tennis ball” OR AB=“sleep position*” OR AB=pillow* OR AB=binder* OR AB=backpack* OR AB=alarm* OR AB=“electrical sensor*” OR AB=“electric sensor*” OR AB=“positional sleeper” OR AB=“positional sleepers” OR AB=band* OR AB=Sona OR posture OR AB=position OR AB=positional OR AB=positioning OR supine OR AB=vibrati* OR AB= “semi-upright” OR AB= “side-lying” OR AB= “back-lying” | 3686727 |
|  | #4 | #1 AND #2 AND #3 | 360 |
| CENTRAL [Date of search: 04/06/2024] | | | |
|  | #1 | “obstructive sleep apnea” OR “Sleep Apnea Syndrome” OR “Sleep Apnea Hypopnea” OR “obstructive sleep apnoea” OR “positional sleep apnea” OR OSAS OR OSAHS OR OSA OR POSA | 7959 |
|  | #2 | Random* | 1427217 |
|  | #3 | “positional therapy” OR “positional treatment” OR “patient positioning” OR “position trainer” OR “position trainers” OR “tennis ball” OR “sleep position” OR “sleep positional” OR “sleep positions” OR pillow* OR binder* OR backpack* OR alarm* OR “electrical sensor” OR “electrical sensors” OR “electric sensor” OR “electric sensors” OR “positional sleeper” OR “positional sleepers” OR band* OR Sona OR posture OR position OR positional OR positioning OR supine OR vibrati* OR “semi-upright” OR “side-lying” OR “back-lying” | 75762 |
|  | #4 | #1 AND #2 AND #3 | 457 |
| Clinicaltrials.gov [Date of search: 04/06/2024] | | | |
|  | Condition/disease | Obstructive Sleep Apnea | 2144 |
|  | Other terms | |  |
|  | Intervention/treatment | Positional Therapy | 129 |
|  | #4 | #1 AND #2 AND #3 | 22 |
|  | Filter | Completed | 11 |
| Google Scholar [Date of search: 04/06/2024] | | | |
|  | With all of the words | obstructive sleep apnea random | - |
|  | With the exact phrase | - | - |
|  | With at least one of the words | positional trainer tennis ball position binder pillow alarm backpack sensor sleeper band posture vibrating vibration "side lying" "back lying" "semi upright" | - |
|  | Total | As per recommendations, only the first 200 records were screened. | 200 |
