# Supplementary material for: Comparative efficacy of sleep positional therapy, oral appliance therapy, and CPAP in obstructive sleep apnea: a meta-analysis of mean changes in key outcomes
Source: Front Med (Lausanne). 2025 Feb 3;12:1517274. doi: 10.3389/fmed.2025.1517274 (PMC11830591; doi:10.3389/fmed.2025.1517274)
Supplement: Supplementary file 2 [file Data_Sheet_1.pdf]

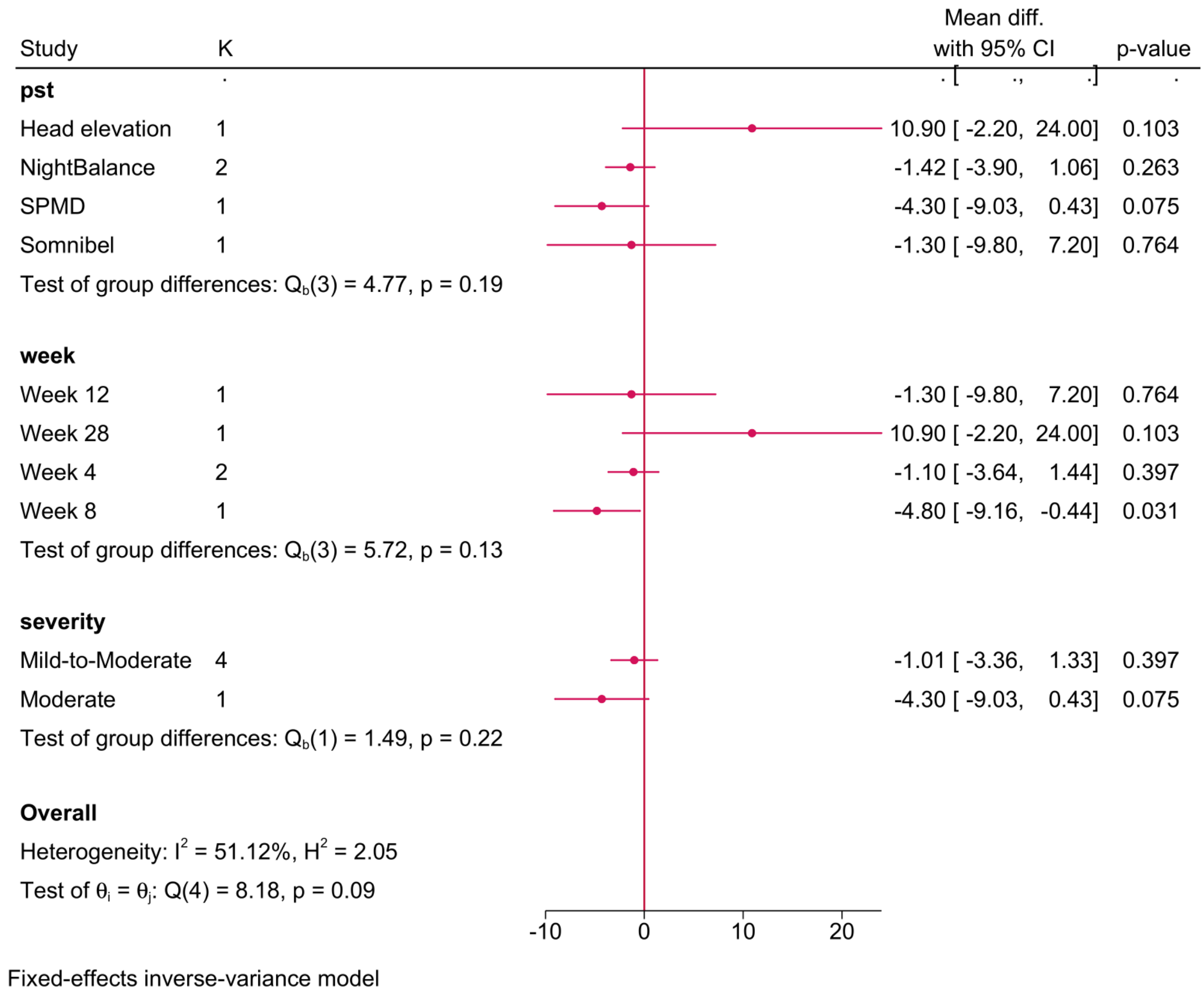

Figure S1. Forest plot showing the difference in the mean change (pre- vs. post-intervention) in AHI score between SPT and placebo, stratified by SPT type, follow-up period, and OSA severity. OSA: obstructive sleep apnea; SPT: sleep positional therapy; AHI: apnea-hypopnea index.

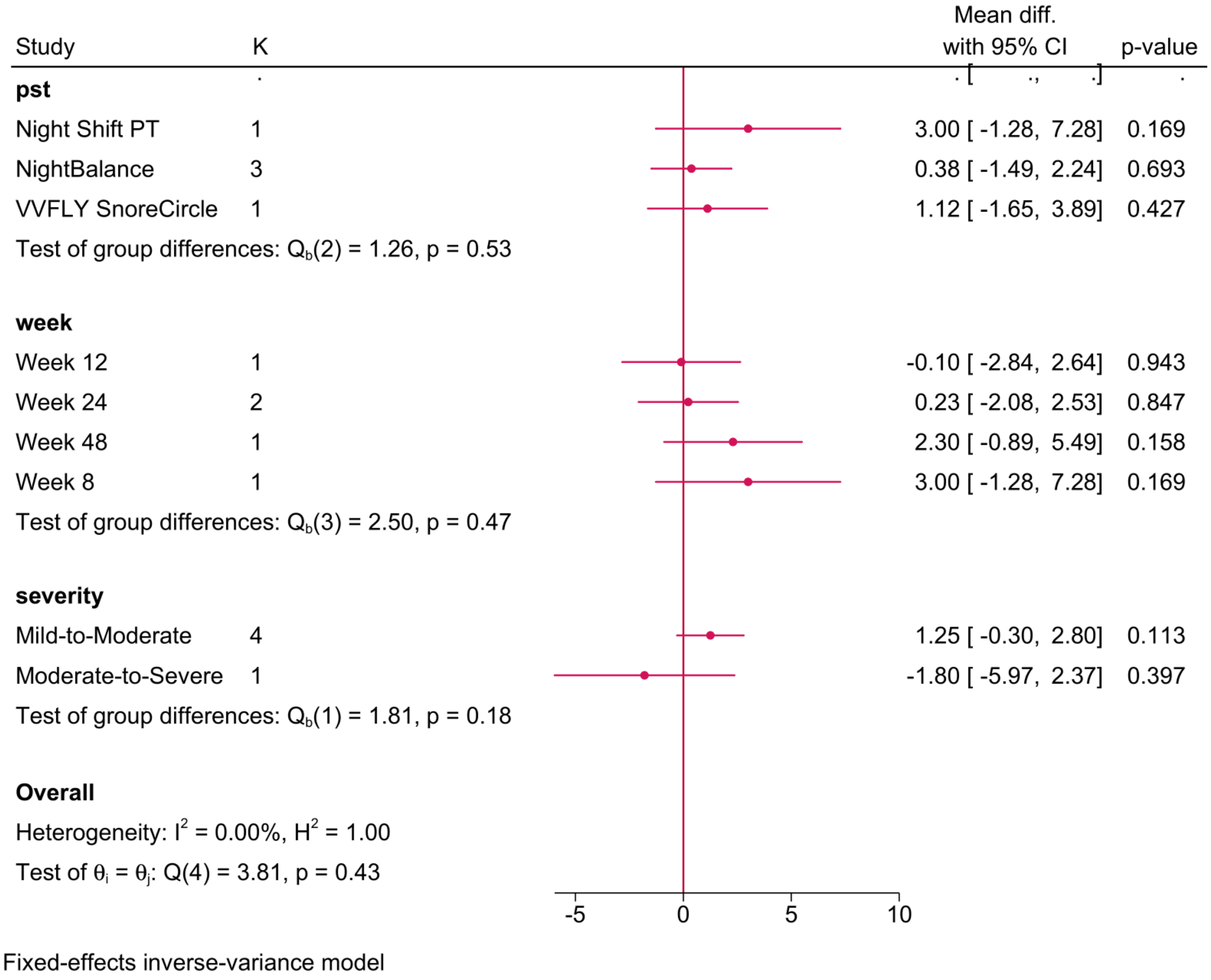

Figure S2. Forest plot showing the difference in the mean change (pre- vs. post-intervention) in AHI score between SPT and OAT, stratified by SPT type, follow-up period, and OSA severity. OSA: obstructive sleep apnea; SPT: sleep positional therapy; AHI: apnea-hypopnea index; OAT: oral appliance therapy.

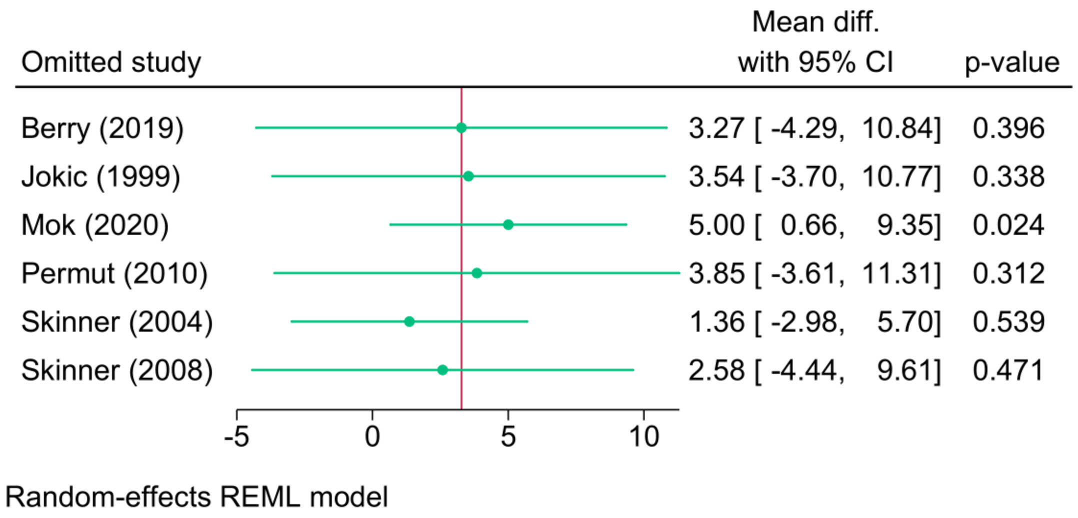

Figure S3. Sensitivity analysis of the difference in the mean change (pre- vs. post-intervention) in AHI score between SPT and CPAP. SPT: sleep positional therapy; CPAP: continuous positive airway pressure; AHI: apnea-hypopnea index.

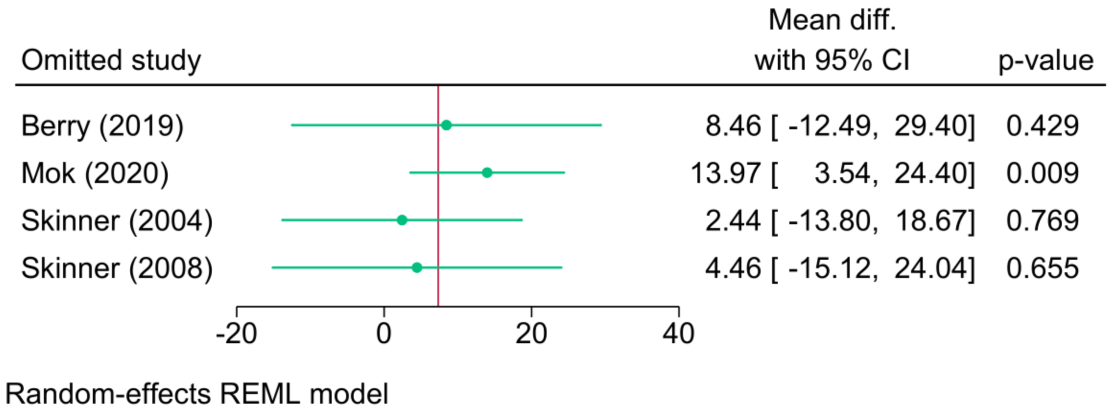

Figure S4. Sensitivity analysis of the difference in the mean change (pre- vs. post-intervention) in AHI score in supine position between SPT and CPAP. SPT: sleep positional therapy; CPAP: continuous positive airway pressure; AHI: apnea-hypopnea index.

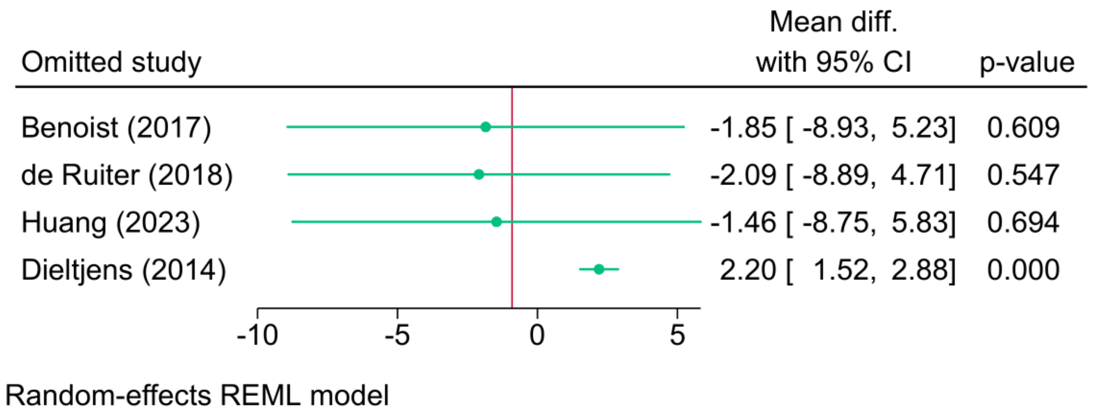

Figure S5. Sensitivity analysis of the difference in the mean change (pre- vs. post-intervention) in AHI score in non-supine position between SPT and OAT. SPT: sleep positional therapy; CPAP: continuous positive airway pressure; AHI: apnea-hypopnea index; OAT: oral appliance therapy.

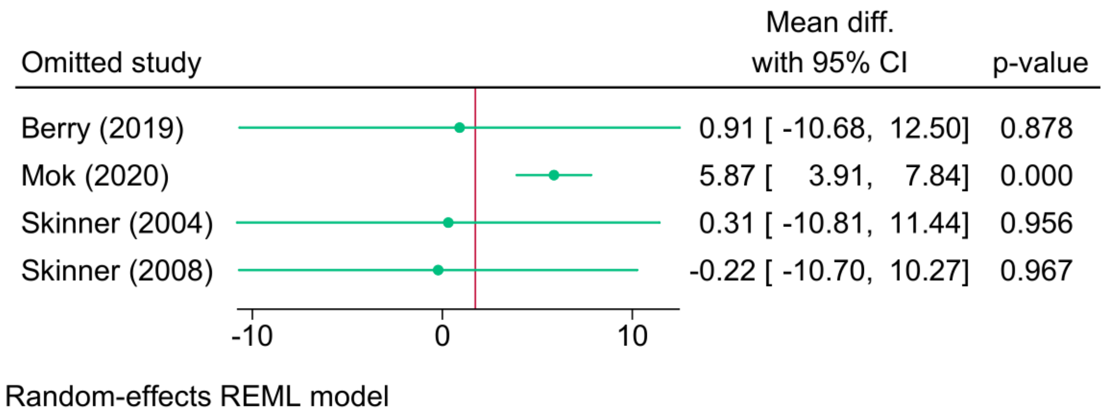

Figure S6. Sensitivity analysis of the difference in the mean change (pre- vs. post-intervention) in AHI score in non-supine position between SPT and CPAP. SPT: sleep positional therapy; CPAP: continuous positive airway pressure; AHI: apnea-hypopnea index.

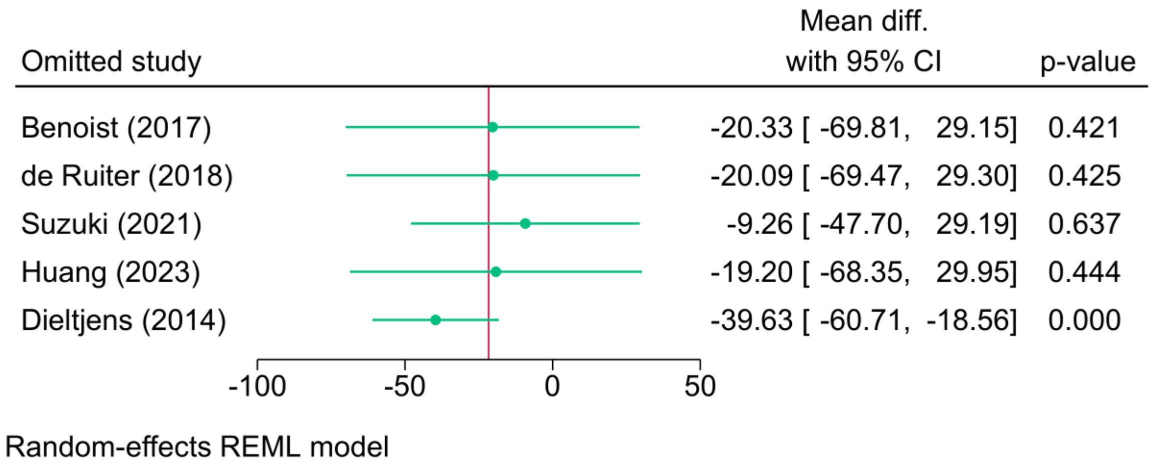

Figure S7. Sensitivity analysis of the difference in the mean change (pre- vs. post-intervention) in TST in supine position between SPT and OAT. SPT: sleep positional therapy; OAT: oral appliance therapy; TST: total sleep time.

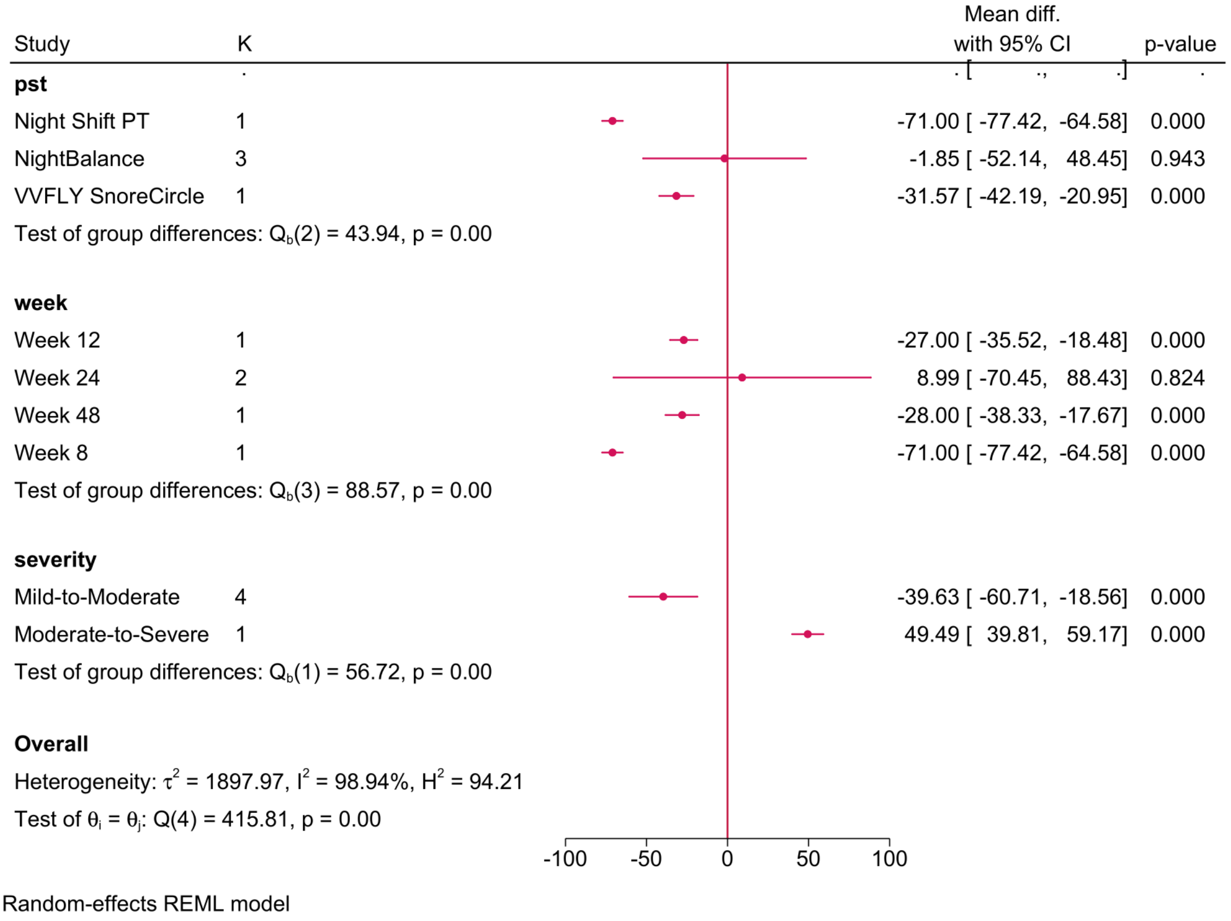

Figure S8. Forest plot showing of the difference in the mean change (pre- vs. post-intervention) in TST in supine position between SPT and OAT, stratified by SPT type, follow-up duration, and OSA severity. SPT: sleep positional therapy; OAT: oral appliance therapy; TST: total sleep time; OSA: obstructive sleep apnea.

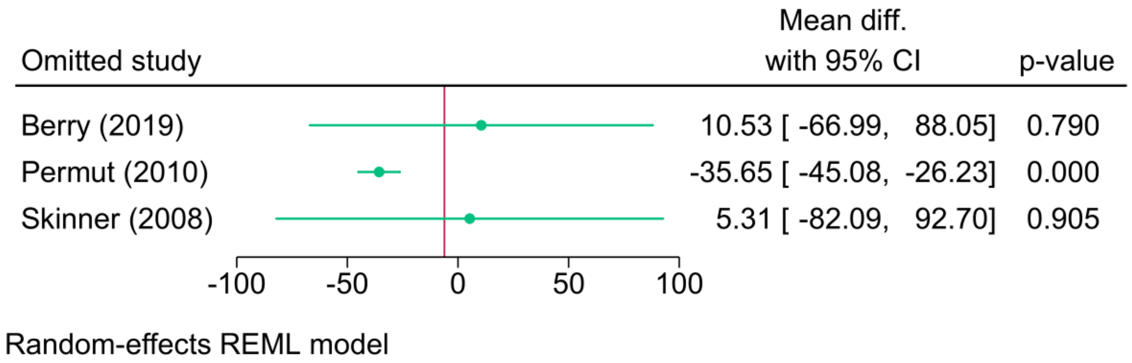

Figure S9. Sensitivity analysis of the difference in the mean change (pre- vs. post-intervention) in TST in supine position between SPT and CPAP. SPT: sleep positional therapy; CPAP: continuous positive airway pressure; TST: total sleep time.

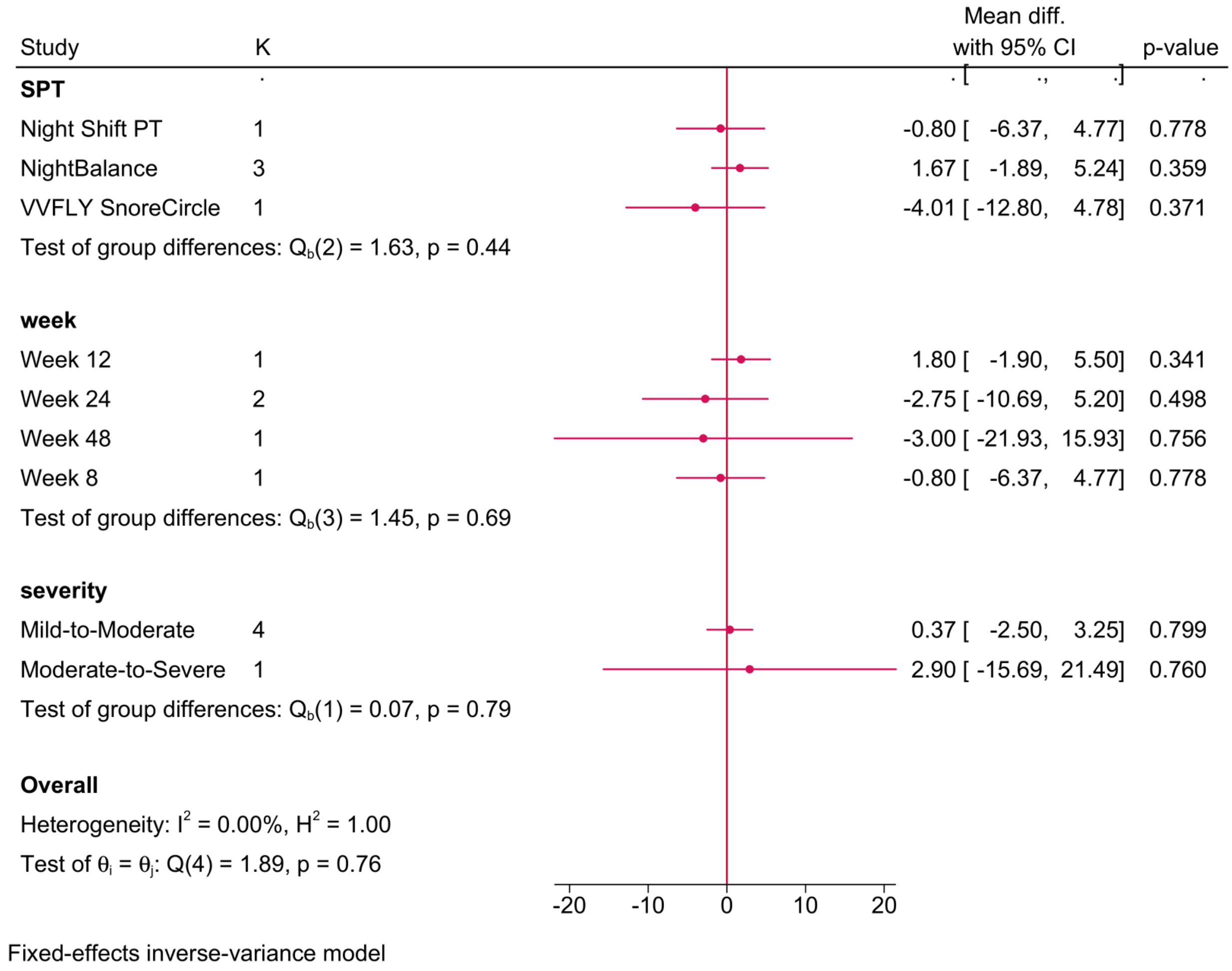

Figure S10. Forest plot showing of the difference in the mean change (pre- vs. post-intervention) in sleep efficiency score between SPT and OAT, stratified by SPT type, follow-up duration, and OSA severity. SPT: sleep positional therapy; OAT: oral appliance therapy; OSA: obstructive sleep apnea.

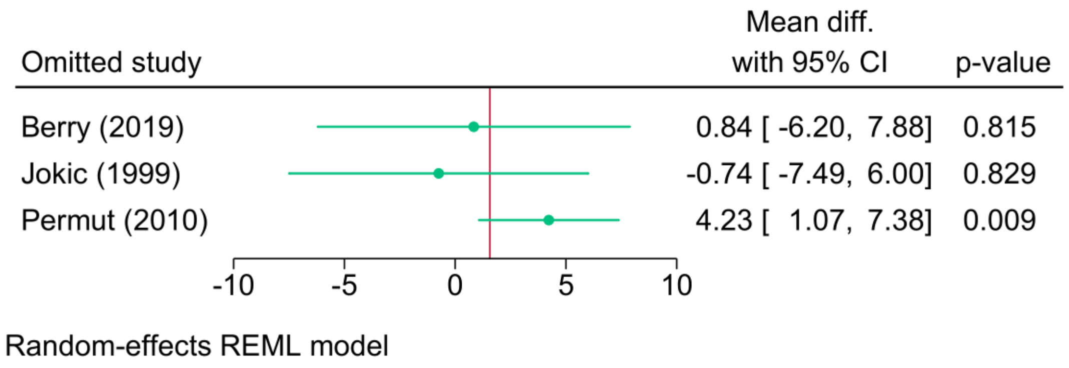

Figure S11. Sensitivity analysis of the difference in the mean change (pre- vs. post-intervention) in sleep efficiency score between SPT and CPAP. SPT: sleep positional therapy; CPAP: continuous positive airway pressure; TST: total sleep time.

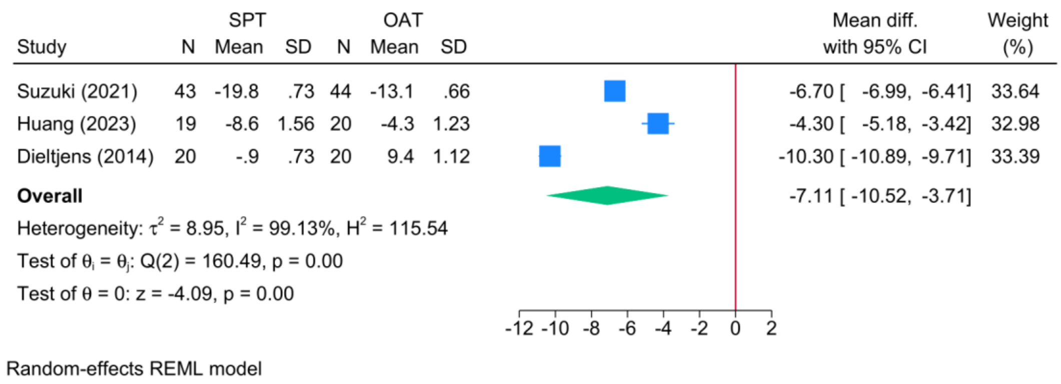

Figure S12. Forest plot showing the difference in arousal index between SPT and OAT. OAT: oral appliance therapy; SPT: sleep positional therapy.

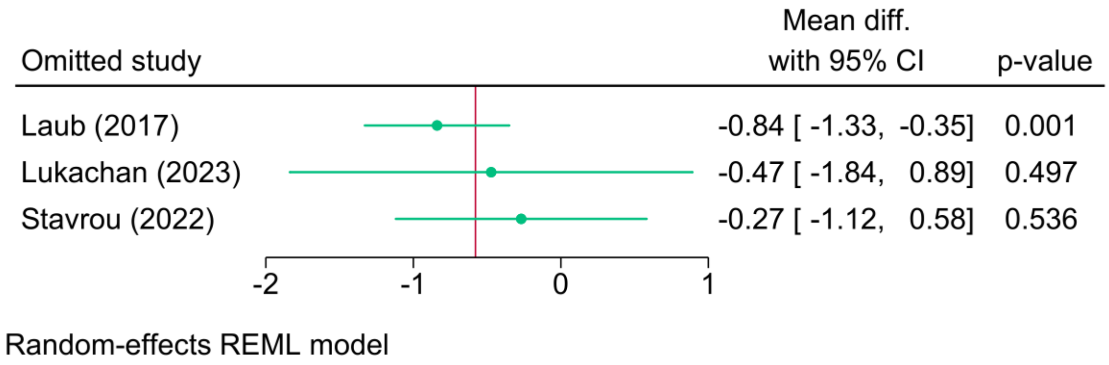

Figure S13. Sensitivity analysis of the difference in the mean change (pre- vs. post-intervention) in mean SaO2 between SPT and placebo. SPT: sleep positional therapy.

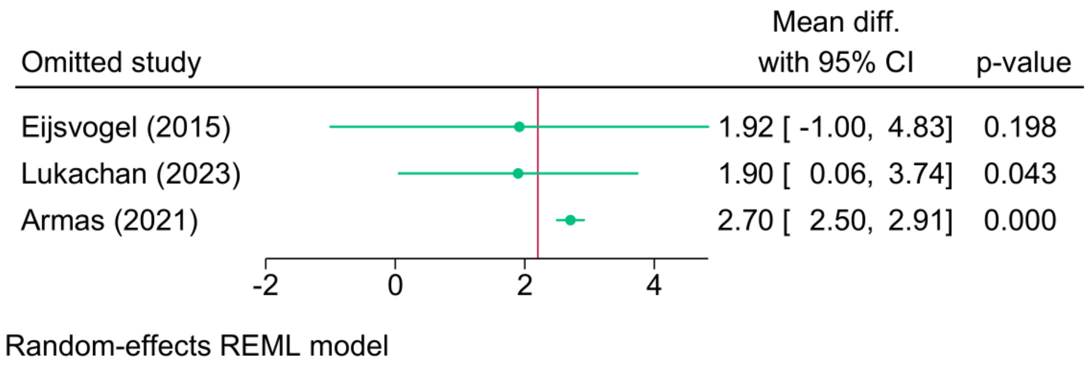

Figure S14. Sensitivity analysis of the difference in the mean change (pre- vs. post-intervention) in ODI score between SPT and placebo. SPT: sleep positional therapy; ODI: O2 desaturation index.

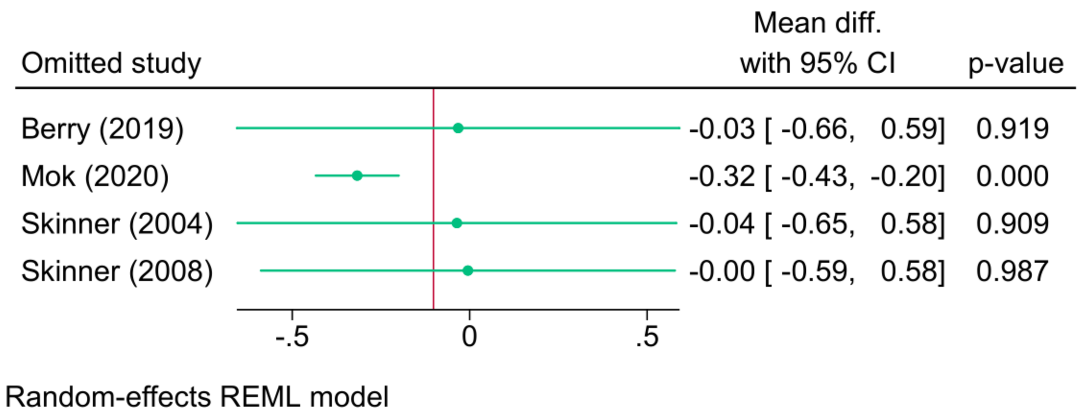

Figure S15. Sensitivity analysis of the difference in the mean change (pre- vs. post-intervention) in FOSQ score between SPT and CPAP. SPT: sleep positional therapy; CPAP: continuous positive airway pressure; FOSQ: functional outcomes of sleep questionnaire.

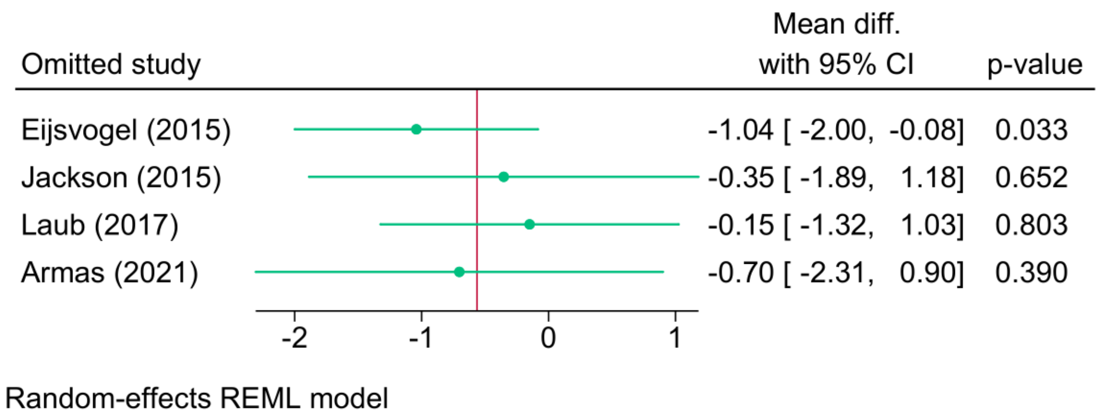

Figure S16. Sensitivity analysis of the difference in the mean change (pre- vs. post-intervention) in ESS score between SPT and placebo. SPT: sleep positional therapy; ESS: Epworth sleepiness scale.

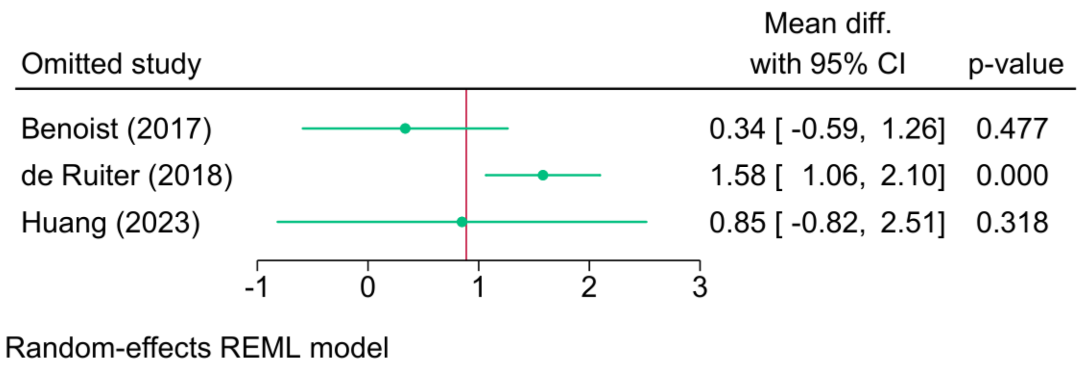

Figure S17. Sensitivity analysis of the difference in the mean change (pre- vs. post-intervention) in ESS score between SPT and OAT. SPT: sleep positional therapy; OAT: oral appliance therapy; ESS: Epworth sleepiness scale.

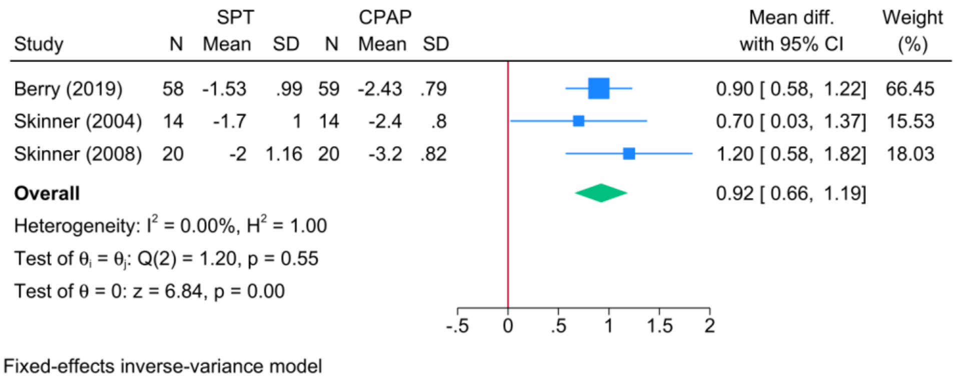

Figure S18. Forest plot showing the difference in ESS score between SPT and CPAP. SPT: sleep positional therapy; ESS: Epworth sleepiness scale; CPAP: continuous positive airway pressure.

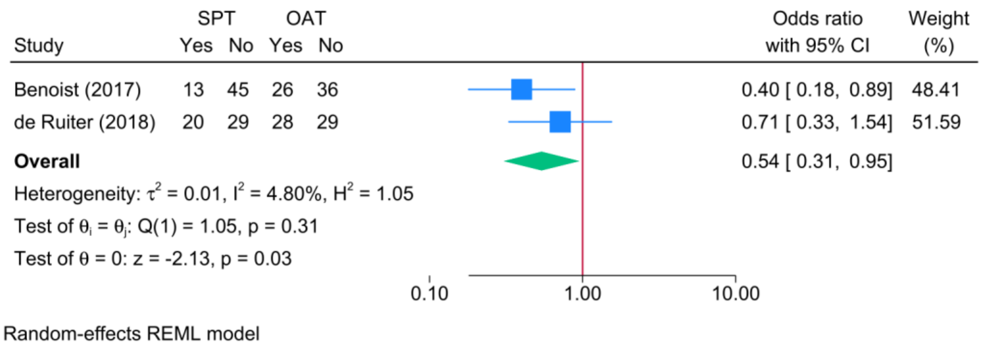

Figure S19. Forest plot showing the difference in the risk of complications between SPT and OAT. OAT: oral appliance therapy; SPT: sleep positional therapy.
